# Matching laboratory and real-world listening environments with ecological momentary assessment improves prediction of speech perception

**DOI:** 10.1121/10.0046844

**Published:** 2026-10-13

**Authors:** Erik Jorgensen, Megan Werner, Autumn Grove, Afton Noll

**Affiliations:** Department of Communication Sciences and Disorders, University of Wisconsin—Madison, Madison, Wisconsin 53704, USA

## Abstract

Laboratory speech-in-noise measures fail to predict real-world listening performance. This study tested whether matching laboratory to real-world environments using self-reported noise sources from ecological momentary assessment (EMA) improves predictions. Speech intelligibility and listening effort were measured in virtual acoustic environments in 51 normal-hearing adults, who then self-rated both via EMA for 1 week. Laboratory intelligibility predicted real-world intelligibility only when laboratory and real-world environments were matched. Effort results were not significant. Findings suggest that environment-specific laboratory testing improves ecological validity of intelligibility measures, though environment matching explained only 1.1% of variance in real-world ratings, with individual differences accounting for substantially more.

## 1. Introduction

A focus of audiology research is understanding and improving real-world outcomes of hearing interventions, particularly in the noisy environments where individuals with hearing loss struggle most. Intervention benefit can be measured as efficacy (benefit under controlled laboratory conditions) or effectiveness (benefit in daily life). Efficacy is typically measured using behavioral speech-in-noise tests, which are clinically efficient but have historically used unrealistic masking noises that bear little resemblance to real-world listening environments (Francis, 2022). Effectiveness has traditionally been measured using retrospective questionnaires, which have well-documented recall biases and do not capture the variability of real-world listening experiences (Wu *et al.*, 2020). Consequently, laboratory and self-report measures often disagree, reflecting a persistent efficacy–effectiveness gap (Wu *et al.*, 2019). Two approaches have emerged to address this gap: virtual acoustic environments that bring ecological realism into the lab and ecological momentary assessment (EMA), repeated surveys that capture subjective listening experiences in daily life.

Virtual acoustic environments enable precise behavioral measurement under realistic acoustic conditions (Neidhardt *et al.*, 2022; Grimm *et al.*, 2019; Weisser *et al.*, 2019; Hohmann *et al.*, 2020). Performance in virtual environments is typically worse than on standard clinical tests, partly because those tests use unrealistic noise, such as speech-shaped noise or multi-talker babble that is non-reverberant, non-spatialized, and dynamically compressed (Best *et al.*, 2015; Mansour *et al.*, 2021b; Miles *et al.*, 2022). Performance in a virtual environment is typically better than in a real-world room, suggesting virtual environments track real-world performance directionally but incompletely (Ahrens *et al.*, 2019; Cubick and Dau, 2016). Virtual environments enable acoustic realism but cannot reproduce all real-world acoustic factors, and behavioral tasks like sentence repetition are themselves not ecological. Non-acoustic factors that shape real-world listening (visual cues, talker familiarity, and situation importance) are often controlled away, leaving uncertainty as to whether performance in virtual acoustic scenes predicts daily-life listening. EMA measures real-world listening performance and has become common in hearing research (Holube, 2020; Schinkel-Bielefeld *et al.*, 2024). It typically involves prompting listeners via smartphone app, many times a day over many days, to, for example, report their listening environment and rate their speech perception. Because EMA captures experiences as they occur, it provides contextualized information with low recall bias (Shiffman *et al.*, 2008), and self-reported EMA intelligibility correlates with behavioral measures under controlled conditions (Wu *et al.*, 2015). However, EMA sacrifices experimental control: Many acoustic and non-acoustic factors influence real-world ratings of listening performance, making it challenging to interpret EMA data with reference to performance under controlled conditions.

Virtual acoustic environments and EMA represent complementary approaches to the same problem but have not been evaluated in relation to each other, raising the central question of whether behavioral performance in virtual environments predicts EMA-measured real-world listening performance. The lab-to-real-world disconnect is often attributed to broad failures of ecological validity, but a more specific hypothesis is that noisy environment mismatch is one key driver—Different types of background noise impose different speech processing demands, and performance in one noisy environment may not generalize to a dissimilar one (Jorgensen, 2025). If so, virtual environments should predict performance in the specific noisy environments they emulate. If non-acoustic factors dominate [e.g., Erber (1975), Jorgensen *et al.* (2025), and Souza *et al.* (2013)], virtual environments may not predict real-world performance regardless of their acoustic similarity.

The purpose of the present study was to test whether laboratory measures of speech intelligibility and listening effort in virtual acoustic environments predict real-world EMA self-reports and whether matching laboratory to real-world environments strengthens this relationship. We addressed two research questions:

(1) To what degree do behavioral measures of speech intelligibility and listening effort in virtual acoustic noisy environments predict subjective ratings of intelligibility and listening effort in real-world noisy environments?
(2) Does matching laboratory environments to real-world environments using self-reported noise sources on EMA strengthen the lab-to-real-world relationship, relative to mismatched or standard speech-shaped noise conditions?

To address these questions, we tested speech intelligibility and listening effort in four archetype virtual environments and a speech-shaped-noise reference in normal-hearing adults who then completed 1 week of EMA.

## 2. Materials and methods

Participants completed laboratory testing across two visits 1 week apart, with EMA completed during the intervening week. Visit 1 included standard audiometry and speech intelligibility assessment; visit 2 included the listening effort assessment. Assessments were split across visits to limit fatigue. This study was approved by the Institutional Review Board at University of Wisconsin—Madison (2023-0710), and all participants provided written consent and received compensation.

### 2.1 Participants

The data presented here were drawn from a larger data collection effort examining aspects of auditory lifestyle (Jorgensen *et al.*, 2026). Participants were 51 young adults [36 female, 13 male, 2 non-binary, mean age (*M*) = 21.2 years, standard deviation by age (SD) = 2.3 years] with typical hearing [pure-tone thresholds of ≤25 dB hearing level (HL) at standard audiometric frequencies]. We focused on young adults to maximize the diversity of qualifying EMA environments, as older adults spend less time in noisy environments and are less likely to complete EMAs in them (Schinkel-Bielefeld *et al.*, 2020; Wu and Bentler, 2012).

### 2.2 Laboratory measurements

To simulate real-world noisy environments, we used the Ambisonics Recordings of Typical Environments (ARTE) database (Buchholz and Weisser, 2019; Weisser *et al.*, 2019), a set of realistic recordings of common real-world listening environments. Each environment contains naturalistic, spatially distributed maskers recorded *in situ* using a 62-channel microphone array. From several hours of recordings in each environment, the developers selected representative segments of ∼2 min. We selected four archetype environments: Church (quiet speech from few talkers), Cafe (speech and mechanical noise), Dinner Party (speech and music), and Food Court (loud multi-talker speech). We chose these environments because prior work established that they are perceptually and acoustically distinct, yielding different speech perception scores and frequency importance functions even at matched signal-to-noise ratios (SNRs) (Jorgensen, 2025), providing an empirical basis for treating them as archetypes representing distinct real-world “cocktail party” listening situations. Speech-shaped noise from the ARTE database was included as a standard reference condition. This speech-shaped noise was not spectrally matched to our speech corpus, and Weisser *et al.* (2019) report its spectrum only graphically. We used it because it serves as the calibration reference for all ARTE environments and has established behavioral characteristics relative to the other environments. Recordings were decoded to eight loudspeakers via a 3rd-order decoder and presented from a circular array in a double-walled sound booth [KEF Q150 speakers (KEF International, Maidstone, UK), Crown XLS 1502 amplifiers (Crown Audio, Harmon International Industries, Stamford, CT), and MOTU i/o 24 interface (MOTU, Cambridge, MA); 44.1 kHz, 32-bit WAV files; flat free-field response 0.16–10 kHz]. Speech-shaped noise was presented as a diffuse field across all loudspeakers using randomized inter-channel phases. The sound levels (dBA) and reverberation times (RT60) are provided in the ARTE database. We additionally calculated speech clarity (C50) from the omnidirectional channel of the room impulse responses, averaged across the 0.5, 1, and 2 kHz octave bands, using the rt60 function in matlab. Sound levels, RT60s, and C50 values for each environment are provided in Table S1 in the supplementary material. Playback levels were calibrated using a 0.5-in. omnidirectional microphone [Brüel & Kjaer type 4189 (Brüel & Kjaer, Naerum, Denmark)]. The ARTE files are normalized such that calibrating playback of the provided speech-shaped noise to 65.9 dBA ensures all environments are reproduced at their original recorded sound pressure levels. Further details about the development and acoustic descriptors (frequency spectra, directional characteristics, temporal envelopes, and modulation spectra) of the ARTE database can be found in Weisser *et al.* (2019).

Speech intelligibility was measured as the SNR at which the participant correctly perceived 50% of target speech (SNR50), using a one-up, one-down adaptive procedure based on the Hearing in Noise Test (Nilsson *et al.*, 1994). Target stimuli were high-fidelity recordings by a female talker of Bamford–Kowal–Bench (BKB) sentences from Miller *et al.* (2025). Target speech tokens were convolved with a single room impulse response (RIR) for each environment from the ARTE database, originally measured by Weisser *et al.* (2019) with the loudspeaker positioned 1.3 m from the microphone array. This source-receiver distance was therefore fixed by the original recordings and held constant across all environments in our stimuli. Target sentences for each environment were calibrated on the reverberant speech signal. For the speech-shaped noise condition, target sentences were presented without reverberation, from the front speaker only. The initial target sentence SNR for each test was −12 dB, with 4 dB step sizes for the first four trials and 2 dB subsequent step sizes. To be considered correct, the participant had to get the target sentence exactly correct. The SNR50 was taken as the mean of the final 17 of 20 trials. Each participant completed a practice test followed by two tests per environment. Target sentences were drawn randomly without replacement. Environment order was random.

Listening effort in the lab was measured using the vocal reaction time (VRT) paradigm (Gustafson *et al.*, 2023; Pals *et al.*, 2015). Monosyllabic words were presented to the participant, and they repeated the word. The time between target offset and participant response onset is the VRT and is the measure of listening effort, with longer VRTs indicating more effort. We used the monosyllabic word corpus from Picou *et al.* (2013). Target words were presented at their estimated real-world SNR based on the function described in Wu *et al.* (2018); values for each environment are provided in Table S1 in the supplementary material. Each test comprised 25 words. The participant first completed a practice in quiet, followed by two tests in each noise in random order. Responses were recorded and saved as audio files. Target offset and response onset times were hand-coded offline by two trained lab members, retaining only clearly audible trials. All trials, correct or incorrect, were included in the analysis (Gustafson *et al.*, 2023).

### 2.3 EMA

Ecological momentary assessments were delivered to participants' own smartphones using the expiwell app every 2 h across a self-selected 16-h daily window (8 surveys/day), with a 15-min random jitter and a 15-min response window. Participants were asked what they were mostly listening to (Nothing, Speech, Music, Nature, Noise, or Something else), whether background noise was present, and if so, to identify the primary and any secondary noise sources (Speech, Music, Machines, Nature, or Something else) and rate background noise loudness (0–10). If listening to speech, they rated speech intelligibility (“How well were you able to understand the speech?”: 0 = not at all well, 10 = perfectly well) and listening effort (“How hard did you have to work to understand the speech?”: 0 = not hard at all, 10 = very hard).

For inclusion in analyses, the participant had to report that they were listening to speech in the presence of background noise. From these, each EMA was categorized into one of the archetype environments to match the virtual environments based on the following criteria:

(1) Church: Only speech in the background, background noise loudness rating equal to or less than the mean background noise loudness rating
(2) Cafe: Most prominent noise either speech or machines but must contain both speech and machines, and no other sounds
(3) Dinner Party: Most prominent noise either speech or music but must contain both speech and music, and no other sounds
(4) Food Court: Most prominent noise must be speech, no music or machine noise but can have other environmental sounds, background noise loudness rating greater than the mean background noise loudness rating

EMA observations that did not fit into one of these categories were not included in the analyses. We categorized real-world environments by noise sources rather than environment type (e.g., an actual cafe or church) because noise source is identifiable via simple self-report in a way that objective acoustic measures cannot and because a location's listening environment can vary across tokens or over time (Jorgensen *et al.*, 2021). We focused specifically on self-report EMA using typical survey questions and response formats given its popularity, logistical simplicity, and instrument-free implementation; objective acoustic measures were not included (see Discussion). All participants completed a practice EMA in the lab to confirm they understood the questions and format. They were not instructed to seek out specific environments or alter their lifestyle and were not told their lab ratings would be compared to their EMA ratings.

### 2.4 Analysis

To characterize environment differences, we fitted linear mixed-effects models with random intercepts for participants to laboratory and EMA data separately, reporting omnibus *F* tests from type III analysis of variance (ANOVA). To test whether environment matching moderates lab-to-real-world prediction, we fitted separate models for speech intelligibility and listening effort with EMA ratings as the dependent variable, lab score (SNR50 or VRT), match condition (matched, mismatched, or speech-shaped noise), and their interaction as predictors. All mixed-effects models were fit to individual-level observations, with random intercepts for participants accounting for non-independence of repeated EMA observations. Each EMA observation was paired with all five lab conditions, enabling a within-participant comparison of predictive validity across match conditions. Lab scores were grand-mean centered. The critical tests were the SNR50 × match condition and VRT × match condition interactions; condition-specific slopes were extracted to characterize the nature of significant interactions. Environment-specific slopes within matched pairs were examined using analogous models with environment replacing match condition. Marginal and conditional $R^{2}$ values were computed to partition variance explained by fixed effects vs individual differences. A limitation is that pairing each EMA observation with all five lab conditions inflates the effective sample size, most acutely for the speech-shaped-noise slope, which is informed entirely by between-participant variation since each participant contributes a single lab value; the resulting standard error therefore overstates the precision of this estimate. The matched and mismatched slopes retain some within-participant variation across environments and are comparatively less affected. We also conducted participant-level correlation analyses independent of this pairing structure. All analyses used r (v4.5.0) with lme4, lmertest, emmeans, and mumin.

## 3. Results

### 3.1 EMA compliance and missing data

Participants completed a mean of 40.7 (SD = 9.3, range = 16–57) EMAs (72.8% compliance). After applying inclusion criteria (background noise present, listening to speech, classifiable listening environment, and not wearing earphones), 296 EMA responses (14.2%) from 48 participants were retained. (Three participants were never in qualifying environments.) The final sample included 123 Dinner Party (41.6%), 70 Church (23.6%), 60 Cafe (20.3%), and 43 Food Court (14.5%) responses. Participants contributed a mean of 6.2 responses (SD = 4.5, range = 1–17) across a mean of 2.5 environments (SD = 1.0).

All participants had complete speech intelligibility data. For listening effort, three participants were excluded because their recorded verbal responses were too quiet for reliable VRT coding, and four had missing conditions due to technical recording errors. Of 11 726 analyzable trials, 1213 (10%) were double-coded with an intraclass correlation of 0.88. Intelligibility was high across environments (range 76.5–95.7% correct), meeting typical criteria for effort measurement above and beyond intelligibility (Winn and Teece, 2021).

### 3.2 Speech intelligibility and listening effort in the lab and real world

Laboratory (behavioral) results are shown in Fig. 1, with speech intelligibility in panel (A) (left) and listening effort in panel (B) (right). Higher SNR50s indicate poorer speech perception and higher VRTs indicate greater effort. Laboratory speech intelligibility varied significantly across environments [ F(4,200)=146.99, p<0.001], improving from Food Court to Dinner Party to Cafe to speech-shaped noise to Church, with a 6.7 dB range between the hardest and easiest environments. Pairwise contrasts showed that all contrasts were significant, except for Church vs speech-shaped noise (see Table S2 in the supplementary material). Laboratory listening effort also differed significantly by environment [ F(4,187)=54.53, p<0.001] and followed the same pattern across environments as speech intelligibility, but differences, particularly between Church, Cafe, and Dinner Party, were small. The significant omnibus test was primarily driven by Food Court requiring significantly longer response times compared to all other environments (see Table S3 in the supplementary material).

**Fig. 1.**
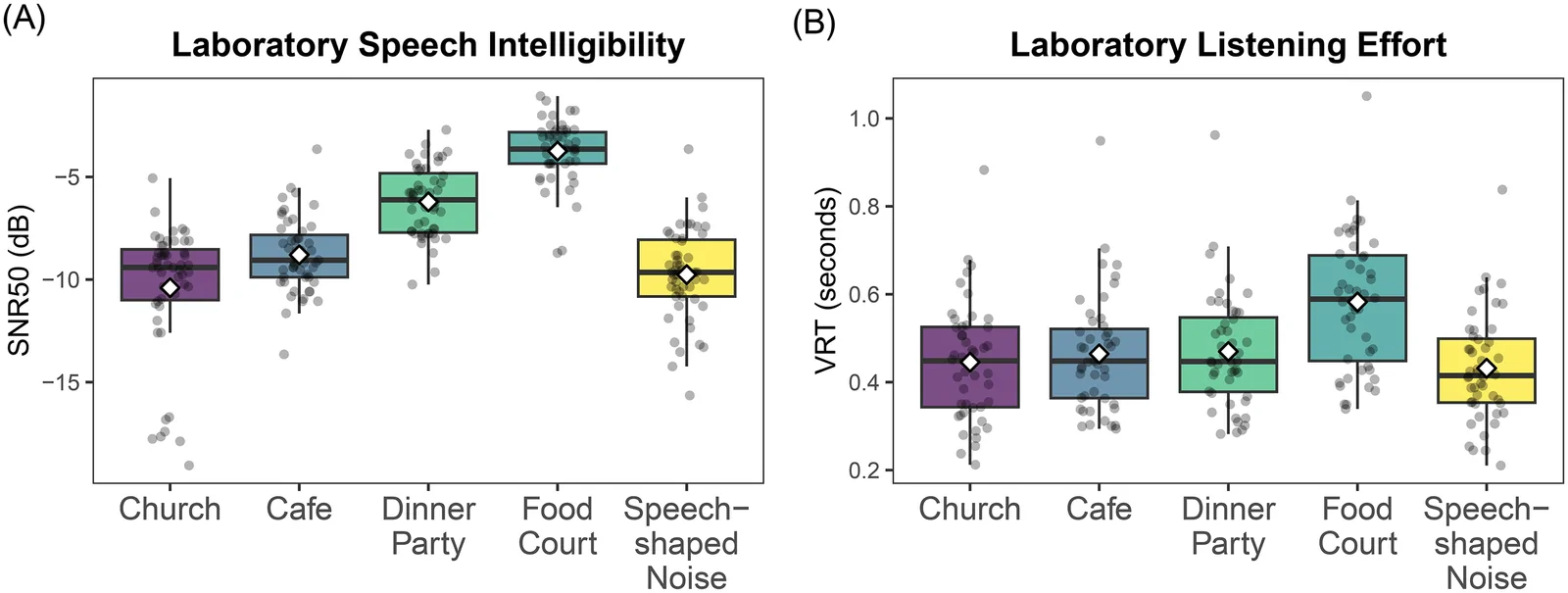
Laboratory speech intelligibility (A) (SNR50, lower = better) and listening effort (B) (VRT, longer = greater effort) across acoustic environments. Dots are raw observations. Box plots show median, quartiles, and range; white diamonds indicate means.

Real-world (EMA self-report) results are shown in Fig. 2, with speech intelligibility in panel (A) (left) and listening effort in panel (B) (right). Lower ratings of speech intelligibility indicate poorer speech perception, and higher effort ratings indicate greater effort. Like laboratory behavioral performance, speech understanding ratings decreased from Church to Cafe to Dinner Party to Food Court. Real-world speech intelligibility ratings also varied significantly across environments [ F(3,291)=7.22, p<0.001], with significant pairwise contrasts between Church and Dinner Party, Church and Food Court, and Cafe and Food Court, but a non-significant difference between Dinner Party and Food Court (see Table S4 in the supplementary material). Despite this significant variation, all environment means fell within a relatively high range (7–9), suggesting generally good self-reported speech perception across listening situations. Real-world listening effort followed the same pattern as in the laboratory, increasing from Church to Food Court (a 2.3-point range) and differing significantly by environment [ F(3,290)=14.23, p<0.001]. Significant contrasts were observed between Cafe and Church, Church and Dinner Party, and Church and Food Court (see Table S5 in the supplementary material). Taken together, in both environment modalities, there was a gradient of difficulty from Church to Cafe to Dinner Party to Food Court, with the starkest differences between Church and Food Court.

**Fig. 2.**
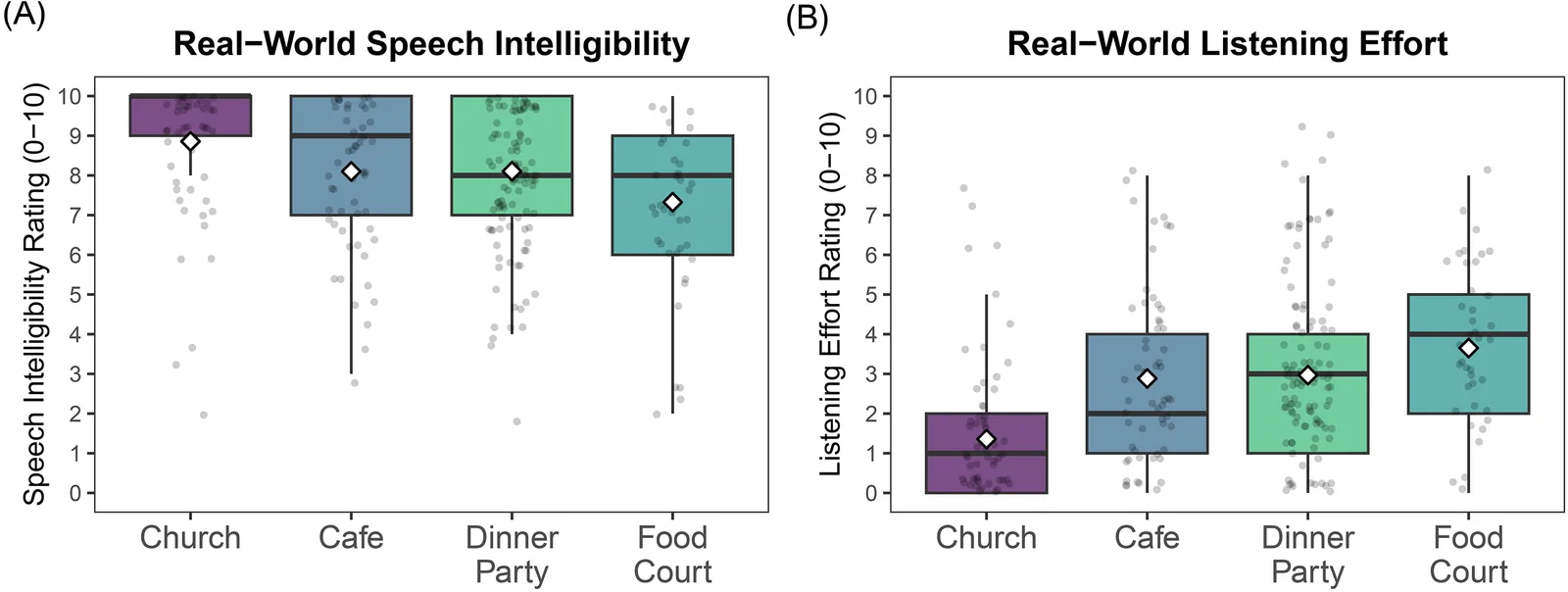
Real-world speech intelligibility (A) (EMA ratings, higher = better) and listening effort (B) (EMA ratings, higher = greater effort) across listening environments. Dots are raw observations. Box plots show median, quartiles, and range; white diamonds indicate means.

### 3.3 Real-world rating predictions by matched and mismatched environments

There was a significant main effect of laboratory SNR50 on real-world speech intelligibility ratings [ F(1,1325)=4.23, p=0.040], but, critically, there was a significant interaction between SNR50 and match condition [ F(2,1318)=9.56, p<0.001], such that laboratory SNR50 predicted real-world ratings, but only when laboratory and real-world environments matched. This is shown in Fig. 3(A), where the mismatched environment and speech-shaped noise slopes are essentially flat, while the matched environment shows a negative slope. Slope estimates and 95% confidence intervals (CIs) are given in the upper section of Table 1 (see pairwise contrasts in Table S6 in the supplementary material). When environments matched, each 1 dB improvement in SNR50 corresponded to a 0.18-point increase in real-world speech intelligibility ratings. In contrast, mismatched environments and speech-shaped noise showed near-zero, non-significant relationships. Despite the statistically significant interaction between match condition and SNR50, lab measures were weak predictors of real-world ratings, explaining only 1.1% of variance in real-world speech perception (marginal $R^{2}=0.011$). Individual differences accounted for much more variability (conditional $R^{2}=0.253$).

**Fig. 3.**
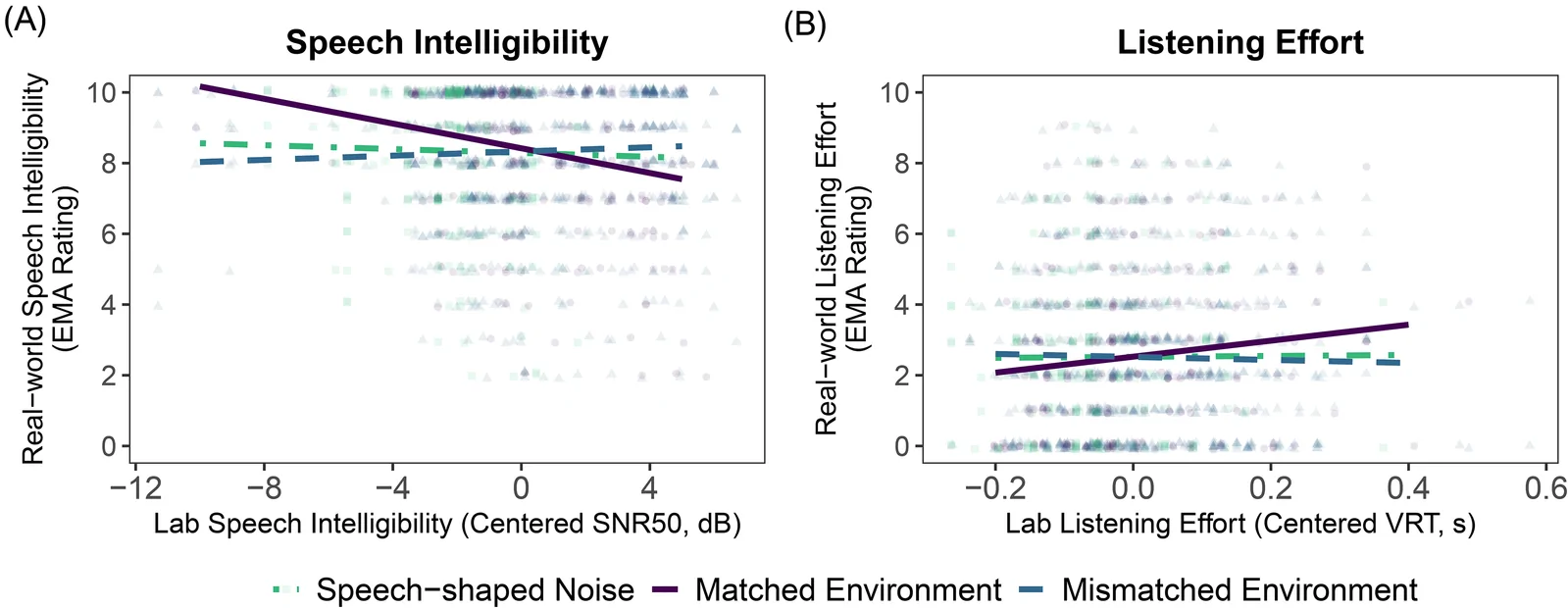
Real-world intelligibility ratings (A) as a function of centered laboratory SNR50 and listening effort ratings (B) as a function of centered VRT, for matched (solid purple, circles), mismatched (dotted blue, triangles), and speech-shaped noise (teal dotted-dashed, squares) conditions.

**Table 1.** Slopes predicting real-world ratings by match condition. Speech intelligibility slopes are per 1 dB increase in SNR50; listening effort slopes are per 1 s increase in VRT.

Outcome	Match condition	Slope	SE	*df*	*t*	*p*	CI_*lower*_	CI_*upper*_
SNR50	Speech-shaped noise	−0.03	0.06	1334	−0.44	0.658	−0.15	0.09
	Matched	−0.18	0.04	1317	−4.03	<0.001	−0.26	−0.09
	Mismatched	0.03	0.02	1331	1.42	0.156	−0.01	0.07
VRT	Speech-shaped noise	0.13	1.48	1218	0.09	0.928	−2.76	3.03
	Matched	2.26	1.24	1237	1.82	0.069	−0.17	4.70
	Mismatched	−0.42	0.78	913	−0.55	0.586	−1.95	1.10

The listening effort analysis did not support the matching hypothesis. There was no main effect of VRT [ F(1,804)=0.58, p=0.445], and the interaction between VRT and match condition did not reach significance [ F(2,1310)=2.45, p=0.087]. Slope estimates and 95% CIs are given in the lower section of Table 1 (pairwise contrasts and *p* values are given in Table S7 in the supplementary material). The matched-environment slope was positive but non-significant ( β=2.26, p=0.069), such that each 0.1 s increase in VRT corresponded to a 0.23-point increase in real-world effort ratings. This pattern is shown in Fig. 3(B). Laboratory measures were weak predictors of real-world listening effort, explaining only 0.3% of variance (marginal $R^{2}=0.003$). Individual differences again accounted for substantially more variability (conditional $R^{2}=0.277$).

To determine whether the matching effect was consistent across environments or driven by particular pairs, we tested interactions between lab scores and environment within matched observations only. Neither interaction reached significance [speech, F(3,214.49)=1.36, p=0.256; effort, F(3,259.74)=1.07, p=0.363], suggesting the matching effect reflects a general tendency rather than being driven by any particular environment pair. Slopes were directionally consistent across all four environments for both outcomes (see Table S8 in the supplementary material). Descriptively, only Food Court yielded a significant individual slope for speech intelligibility ( β=−0.51, p=0.012), consistent with its larger spread of behavioral scores providing greater statistical sensitivity, though this should be interpreted cautiously given the non-significant omnibus test.

### 3.4 Individual differences

The low marginal $R^{2}$ values indicate that lab performance and match condition accounted for little total variance in real-world ratings, while the larger conditional $R^{2}$ values reflect substantial between-participant variability. To characterize this, we computed participant-level Pearson correlations between lab performance and mean EMA ratings across matched environments, restricted to participants contributing data in at least two environments ( n=37 for speech intelligibility, n=34 for listening effort, the latter reflecting VRT exclusions). Participant-level correlations were significantly negative for speech intelligibility [mean r=−0.36, SD=0.69; t(35)=−3.12, p=0.004], indicating that better laboratory performance predicted higher real-world intelligibility ratings on average. Correlations were significantly positive for listening effort [mean r=0.51, SD=0.66; t(31)=4.32, p<0.001], indicating that more effortful laboratory performance predicted higher real-world effort ratings on average. The large SDs relative to the means indicate considerable heterogeneity across individuals, suggesting that while the average lab–EMA correspondence is meaningful, its strength varies substantially across listeners. A parallel between-participant analysis correlating each participant's speech-shaped-noise SNR50 and VRT with their mean EMA speech intelligibility and effort ratings, respectively, found non-significant correlations for both outcomes [ r=−0.23, t(46)=−1.63, p=0.111; r=−0.04, t(43)=−0.27, p=0.787].

## 4. Discussion

The central finding of this study is that matched lab-to-real-world environments using self-reported noise sources from EMA significantly predict speech intelligibility, but mismatched environments and speech-shaped noise do not. The difference in slopes between matched, mismatched, and speech-shaped noise in predicting real-world ratings was meaningful; mismatched and speech-shaped noise showed essentially no predictive power, whereas matched environments showed that for every 1 dB increase in SNR50, real-world ratings decreased by about 0.2. In the lab, it has been shown that different acoustic environments place differential demands in different frequency regions and may require different listening strategies to achieve optimal performance (Jorgensen, 2025). We have extended this finding to the real world: Not only do EMA ratings across environments follow the same pattern of speech intelligibility as the lab, but lab performance only predicts real-world performance when the environments have similar noise sources. Importantly, speech-shaped noise was not predictive of real-world ratings, providing further evidence that the ecological validity of laboratory measures is critical to consider when extrapolating to real-world experience. This is consistent with prior work showing that traditional speech-in-noise tests are not strongly related to real-world performance (Miles *et al.*, 2022; Mansour *et al.*, 2021b; Best *et al.*, 2015) and extends it by showing that emulating real-world environments improves those predictions. Environment-specific slopes were directionally consistent across all four matched pairs, with no significant differences between environments, supporting a general matching effect.

The listening effort analysis did not support the matching hypothesis: The VRT × match condition interaction did not reach significance ( p=0.087), and the matched-environment slope was non-significant ( p=0.069). A likely contributor is that VRTs were presented at fixed, estimated real-world SNRs, which were positive for Church, Cafe, and Dinner Party, yielding similar VRTs across these three environments in the lab. This attenuated individual differences in effort relative to the variability observed on EMA. Future work might address this by presenting effort stimuli at individualized SNRs. Listening motivation and situational importance, which can modulate effort (Jorgensen *et al.*, 2025), likely differed between and within laboratory and real-world contexts in ways we could not assess. Motivation may be more stable in the lab, where participants were compensated and instructed to respond on every trial, whereas in the real world, listeners may invest effort independently of the environment, depending on a conversation's importance. This variability could also partly explain why matching improved intelligibility but not effort prediction.

Effect sizes were small; matching explained only 1.1% of variance in speech intelligibility and 0.3% in listening effort. Individual differences accounted for far more variability, though participant-level correlations between lab performance and mean real-world ratings were significant for both outcomes, with the expected direction observed in the majority of individuals. In contrast, the parallel speech-shaped noise correlations were non-significant, converging with the mixed-model finding that speech-shaped noise lacks predictive validity. Still, the total variance explained was ∼25%, suggesting many unaccounted factors influenced ratings.

On average, participants did not encounter large numbers of qualifying noisy environments. Participants were not prompted to seek out or identify specific environments, and so qualifying observations occurred at whatever rate participants encountered them naturally. A study designed specifically to maximize density for this question could likely achieve more observations with less participant burden, though at some cost to naturalistic sampling. Additionally, our matching scheme was deliberately coarse. We evaluated whether self-report EMA alone, using typical sampling methods and questions, sufficed to improve lab-to-real-world prediction. That even this coarse matching yielded significant lab-to-real-world relationships, where mismatched conditions did not, raises the possibility that more precise matching incorporating objective acoustic measures such as sound levels, SNRs, or scene classifiers could strengthen this correspondence (Borschke *et al.*, 2024; von Gablenz *et al.*, 2026; Mansour *et al.*, 2021c; Schinkel-Bielefeld *et al.*, 2024). Currently available objective measures, however, face limitations for this task: Distinguishing the categories used here from recordings or hearing aid classifiers remains an open problem, as current classifiers would likely label all four environments as “speech-in-noise” (Borschke *et al.*, 2024; von Gablenz *et al.*, 2026). Estimating SNR requires reliable source separation, which is technically challenging in multi-talker environments and requires specialized equipment (Wu *et al.*, 2018; Mansour *et al.*, 2021a), and even similar SNRs or sound levels can yield different speech outcomes across environments (Jorgensen, 2025; Best *et al.*, 2015). Critically, even perfect acoustic matching ignores non-acoustic factors known to affect real-world listening. Laboratory behavioral measures and EMA self-report also capture fundamentally different aspects of listening: Behavioral measures assess perceptual sensitivity using clinical materials that emphasize lexical and syntactic content over the prosodic and discourse-level cues present in natural conversation (Beechey, 2022), while EMA captures subjective experience embedded in a social and situational context. This difference may partly explain the modest lab-to-real-world correspondence observed here, though direct comparisons of clinical and conversational speech recognition remain lacking. Self-reported intelligibility is valid under controlled and guided conditions (Wu *et al.*, 2015; Mansour *et al.*, 2021c), but its accuracy in daily life is unknown, and direct comparisons between SNR50 and self-reported ratings under identical conditions were not obtained here. Further, the SNR50, while advantageous for revealing individual differences, is a different measure than intelligibility ratings and may not be particularly relevant to daily life, as listeners can modify, disengage from, or leave a conversation before tolerating 50% intelligibility. The small variance explained by noise source matching underscores that real-world listening is shaped by a constellation of factors no single method can fully capture, and that behavioral, self-report, and acoustic approaches remain necessary and complementary.

## Supplementary Material

See the supplementary material for pairwise comparison tables for all laboratory and real-world environment contrasts.

## Data Availability

The data that support the findings of this study are openly available in the Open Science Framework at https://osf.io/b6ewt/overview?view_only=d98f5266c5034bdd92e67494eb103b6a.
